# Close encounters on a micro scale: microplastic sorption of polycyclic aromatic hydrocarbons and their potential effects on associated biofilm communities

**DOI:** 10.1186/s40793-025-00747-w

**Published:** 2025-07-08

**Authors:** Jessica X. Song, Brittan S. Scales, Minh Nguyen, Emelie Westberg, Bartosz Witalis, Barbara Urban-Malinga, Sonja Oberbeckmann

**Affiliations:** 1 Federal Institute for Materials Research and Testing (BAM), Unter Den Eichen 87, 12205 Berlin, Germany; 2 Leibniz Institute for Baltic Sea Research (IOW), Seestraße 15, Rostock, 18119 Germany; 3 IVL Swedish Environmental Research Institute, Valhallavägen 81, 114 28 Stockholm, Sweden; 4 National Marine Fisheries Research Institute (NMFRI), Ul. Kołłątaja 1, 81-332 Gdynia, Poland; 5 Present Address: Montana State University, Culbertson Hall, 100, Bozeman, MT 59717 USA

**Keywords:** Plastic pollution, PAH, Microbiome, Emerging contaminants, Aquatic biofilms

## Abstract

**Background:**

Within systems as dynamic as the aquatic environment, it is crucial to address the impacts of an ever-growing network of emerging pollutants at their intersection. With previous research having demonstrated the capacity of microplastics (MPs) to sorb persistent organic pollutants, we ask in our study how different plastic polymers that are found throughout aquatic systems interact with polycyclic aromatic hydrocarbons (PAHs) and how this intersection of pollutants might impact the bacterial communities that form on MP surfaces. We performed an in situ incubation experiment at different sites along the Baltic Sea coast and through a PAH and 16S amplicon analysis, we investigated the sorption patterns of different substrates and their potential impacts on associated biofilm communities.

**Results:**

PAH sorption patterns of polyethylene (PE), polystyrene (PS), and aquaria stone were found to be dictated predominantly by substrate type and secondly by incubation site. While PE showed a general positive trend of sorption, stone rather leached PAHs into the environment, whereas the PAH levels of PS remained relatively unchanged following incubation. These sorption patterns correlated significantly with the composition of biofilm communities observed on all three substrate types after a 6-week incubation period. Strong correlations between specific PAHs and bacterial taxa indicate a direct relationship between these factors. Elevated levels of specific 3- and 4-ring PAHs on PE and PS coincided with higher proportions of specific taxa reportedly capable of hydrocarbon utilisation as well as a reduced diversity among biofilm communities.

**Conclusion:**

The findings in our study highlight the importance of investigating contaminants such as MPs holistically, including any associated substances, to fully understand how they impact surrounding ecological systems as they traverse the different compartments of the aquatic ecosystem.

**Supplementary Information:**

The online version contains supplementary material available at 10.1186/s40793-025-00747-w.

## Background

A list of more than 40,000 contaminants of emerging concern have been identified as of 2018 and continues to grow with new entries added every day [1]. Defined as chemicals or microorganisms that pose a risk to human health and surrounding ecosystems, many of these contaminants fall under the category of persistent organic pollutants (POPs) [2, 3]. In recent years, this list has additionally come to comprise microplastics (MPs) and their transformation products due to their pervasive occurrence in nature [1, 4].

Particularly in environments as dynamic as aquatic ecosystems, emerging contaminants cannot be considered singularly when attempting to understand their impacts and must be seen as a component within a more complex and interconnected system. This entails exploring not only the interactions between pollutant and environment but also any interactions between the different pollutants themselves. Due to their interactive nature, MPs are perhaps the prime example of this axiom. In seawater, these normally inert materials gain surface charges that result in the sorption of materials from the surrounding water column [5]. Circulating freely throughout different ecosystems, MPs can accumulate rich polymeric matrices from the environments to which they are exposed, acting essentially as a passive sampler. This pertains not only to the sorption of complex organic compounds but a wide range of other persistent pollutants as well [6, 7]. A particular point of concern is the capacity of MPs to sorb (absorb or adsorb) polycyclic aromatic hydrocarbons (PAHs) [8–13]. Formed through both biological and anthropogenic processes, PAHs are pervasive throughout aquatic environments and pose a risk to the health of humans and aquatic life due to their reportedly toxic, mutagenic, and carcinogenic nature [14–16]. Longstanding concerns over the widespread occurrence of PAHs throughout aquatic environments rise additionally from the threat they pose to surrounding ecological systems [14, 17].

At the foundation of many aquatic ecosystems are heterogenous communities of microorganisms, acting as key players in important biogeochemical processes that maintain healthy ecosystem functioning [18]. Being ubiquitous in nature, microorganisms encounter and interact regularly with pervasive contaminants. These substances, in turn, can alter their function as system regulators and threaten the overall health of ecological systems [14, 19, 20]. Exposure to elevated levels of PAHs, such as after an oil spill or within highly anthropogenic environments, has been shown for instance to alter microbial community composition [17, 19, 21]. On the surface of MPs, the interplay between emerging contaminants and microbial communities are concentrated. Within the complex scaffolds of insoluble pollutants and organic matter that envelop these hardy substrates, the growth of a wide diversity of microbial assemblages is supported [5, 22]. There exist numerous studies dedicated to understanding the ecology of biofilm communities on MPs in aquatic ecosystems [23–25]. Generally, these communities are found to be shaped more strongly by biogeographical and seasonal factors than by the polymer itself, although certain taxa have been suggested to demonstrate properties of plastic specificity [25–28]. As MPs are often found accompanied by complex chemical loads in the environment, however, investigations of these substrates and the characteristics of their biofilms cannot be reduced only to the polymer itself. While there exists a few of studies on the effects of MPs and other pollutants on their associated biofilms, this avenue of research remains largely overlooked and underexplored [11, 29, 30]. Early findings have suggested that certain POPs, such as plastic additives or specific PAHs, can significantly influence the composition of MP biofilms [31–33]. Fernández-Juárez and colleagues tested the effect of different plastic additives on a consortium of marine bacteria and found fluoranthene, a short-chain PAH, to significantly suppress bacterial growth [33]. Hypotheses have also been drawn on the putative selection of hydrocarbon-degrading Mycobacterium on MP in the North Sea by anthracene, a PAH used in the dyes added to plastics. While these findings provide an important foundation to our understanding of combined contaminant effects on microbial communities, more systematic field investigations into the relationship between MPs and PAHs in nature, and their subsequent effects are still needed. Although chemical sorption and biofilm formation are phenomena also observed of many natural substrates in the environment, MPs differ in the way that they are highly durable and dispersible [34]. This results thus in the risk of a prolonged persistence and wider circulation of highly concentrated hotspots of emerging contaminants throughout the environment.

We aim, therefore, to address the intersection of pollutants within aquatic environments and the potential impact of their interactions on surrounding ecosystems. The objective of this work was specifically to investigate the potential interplay between MPs, PAHs, and associated bacterial biofilms. Within the drainage basin of the Baltic Sea, an in situ incubation experiment was conducted over a period of 6 weeks to observe the sorption of PAHs on MPs and elucidate the role these hydrocarbons might play in shaping MP biofilm communities. We first assessed the PAH sorption patterns of different polymer types (polyethylene and polystyrene) and compared them to a non-plastic control (aquaria stone). The sorption patterns observed of the different substrate types were then compared against the dynamics of MP biofilm communities detected through a 16S amplicon analysis to elucidate their effects on community diversity and composition. We hypothesised that different substrate types would exhibit distinctive patterns of PAH sorption that might in turn play a role in shaping associated biofilm communities. We aim with our findings to broaden our understanding of the interplay between different emerging contaminants and microbial biofilms in surface coastal waters.

## Methods

### Site description

**Fig. 1:**
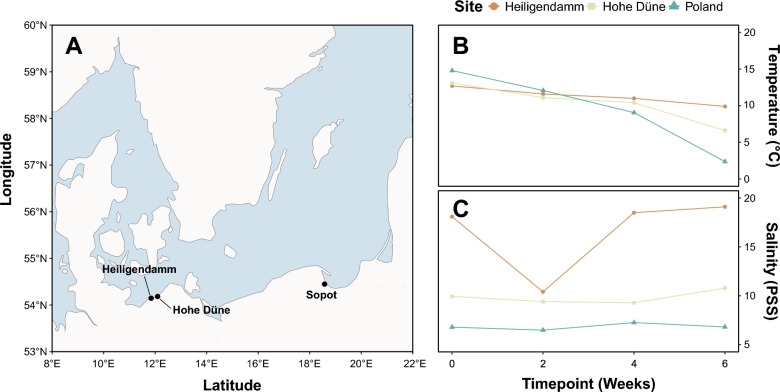
Map showing the location of the incubation experiment alongside recorded abiotic conditions. The three incubation sites were situated along the German (Heiligendamm and Hohe Düne) and Polish (Sopot) coast within the Baltic Sea basin (**A**). Temperature (**B**) and salinity (**C**) of surface waters were recorded at each site throughout a 6-week incubation period between October and December 2018

An incubation experiment was performed in situ at three different sites along the coast of the Baltic Sea (Fig. 1) between October and December 2018. The first two sites were located in the Mecklenburg Bight, situated off piers in Heiligendamm (54°08′44.9′′N 11°50′35.5′′E) and Hohe Düne (54°10′58.8′′N 12°05′42.0′′E) in Germany. Separated by roughly 18 km of coastline, these two sites punctuate the transition zone between the North and Baltic Seas and are marked by regular exchanges of saline marine waters and riverine freshwater inputs [35]. Due to the fluctuating atmospheric conditions that characterise the region, major inflows periodically precede periods of extreme salinity and upwelling of stagnant water masses from deeper layers of the Baltic Proper basin, resulting in high short-term variability. Generally, however, the values range between 14 and 19 PSU, with the salinity maxima typically observed in autumn and winter [36]. The third site, in contrast, is situated within the inner basin of the Gulf of Gdańsk, off the coast of Sopot in Poland (54°26′52.2′′N 18°34′34.2′′E). This site is partially sheltered and receives strong freshwater influences from the Vistula River—the second largest of nine major rivers that feed into the Baltic Sea [37]. Characterised by constant stratification, these waters are composed of saline North Sea inflows along its deeper layers and blanketed by riverine inputs of freshwater on the surface, resulting in a stable salinity gradient [38, 39]. As such, surface waters here are reported to have a salinity of approximately 7–8 PSU [38, 40].

### Experimental setup and sample collection

The incubation experiment was performed using three particulate substrates. Pre-production polyethylene (PE, ExxonMobil™ HDPE HTA 108) and polystyrene (PS, BASF Polystyrole 143E) pellets with an average diameter of 3 mm and densities of 0.961 g/cm^3^ and 1.04 g/cm^3^, respectively, were chosen as synthetic surfaces [28]. As a non-plastic control, similarly sized stone pellets with an average diameter of 2 to 3 mm were used. Stones utilised in the experiment were processed pellets typically used in aquaria and were not sourced from nature.

In situ incubation was carried out using custom-made, metal mesh containers (500 μm mesh size), each enclosing 100–120 particles of a single substrate type. Mesh containers were set up in triplicates for all types at each of the three sites, amounting to a total of nine mesh containers per site. Each set of the triplicates prepared was then secured laterally along a piece of rope, which was fastened to a pier or buoy and anchored in place by a heavy chain. Mesh containers were incubated at an approximate depth of 0.5 to 2 m.

Sample collection for 16S amplicon analysis was performed at 2-, 4-, and 6-week intervals, during which time one container of each substrate type was retrieved. 20 particles were collected from each container and rinsed thoroughly with 0.22 µm-filtered water from the site (Millipore Express® PLUS membrane filter (GPWP04700), ø 47 mm, Merck KGaA, Darmstadt, Germany) to remove any loosely attached planktonic material from particle biofilms. Particles were then placed into 1.5 mL sterile microcentrifuge tubes and flash frozen with liquid nitrogen. All samples were stored at − 80 °C until further processing. For PAH analysis, only particles were sampled at week 0 and week 6, stored in sterile 50 mL tubes, and kept at − 20 °C until processing.

To compare between biofilm and waterborne communities, surrounding waters were also sampled at the beginning of the experiment (week 0) as well as after each incubation period (weeks 2, 4, 6). Three litres of surface water were collected at each site and serially filtered onto 3.0 µm and 0.22 µm filters (polycarbonate Isopore™ membrane filter (TSTP04700), ø 47 mm, Merck KGaA, Darmstadt, Germany) to assess both particle-associated and free-living fractions of waterborne communities, respectively. Filter membranes were then transferred into sterile microcentrifuge tubes and flash frozen. All samples were stored at − 80 °C until further processing.

Surface water temperature and salinity were measured at each site with a portable meter (SonTek CastAway-CTD, YSI Incorporated, Ohio, USA) at the beginning of the experiment and following each incubation period.

### DNA extraction and amplicon sequencing

DNA extraction was performed on pre-incubated, blank particle controls (20 × 3 sites × 3 replicates per type), incubated particles (20 × 3 sites × 3 replicates per type), and water samples (3 sites × 3 – 4 replicates per fraction) according to a previously described protocol (Scales et al., 2021). 700 μl of Tris/Saline/EDTA (TSE) buffer (1 mM EDTA; 50 mM Tris; 6.7% sucrose; pH 8) and 19 μl of lysozyme (10 mg/ml) were added with each sample to a reaction tube and incubated at 37 °C for 1 h. 74 μl of Tris–EDTA (250 mM EDTA; 50 mM Tris; pH 8) and 44 μl of SDS-Tris–EDTA (20 mM EDTA; 50 mM Tris; 20% (w/v) SDS; pH 8) were then added to each tube and incubated at 50 °C for another hour. Following incubation, the mixture was centrifuged at 8,000 × g for 10 min (Eppendorf 5417R, Eppendorf AG, Germany) and the supernatant transferred to a new sterile tube.

1/10 volume of 5 M of NaCl mixed with 1 volume phenol–chloroform (1:1) was added to the reaction tube and centrifuged once more at 8000 × g for 10 min. After transferring the supernatant to a new tube, an equal volume of − 20 °C 100% isopropanol was added to the reaction tube, and the mixture incubated at − 20 °C overnight. On the following day, the reaction tube was centrifuged at 10,000 × g for 20 min at a temperature of 4 °C. The supernatant was then discarded, and the pellet rinsed with 500 μl of 75% ethanol. This step was repeated twice for a total of three washes. After the washing step, the DNA pellet was air-dried and resuspended in 50 μl of PCR-grade water. Total DNA yield was measured with a NanoDrop spectrophotometer (NanoDrop, Kisker, Germany). Reagent blanks and empty microcentrifuge tubes were also prepared as extraction controls.

Library preparation and amplicon sequencing were performed by LGC Genomics GmbH (Berlin, Germany). The V3-V4 regions of 16S rRNA genes were amplified using the Pro341Fadapt (5′-CCTACGGGNBGCASCAG-3′) and Pro805Radapt (5′-GACTACNVGGGTATCTAATCC-3′) [41] primers. Sequencing was carried out using the Illumina MiSeq System (2 × 300 bp, V3 chemistry), following which adapters and primers were removed from resulting reads.

### Read processing

Quality control of demultiplexed paired-end reads was performed on the QIIME 2™ platform v2024.5 [42]. Reads were first denoised and merged using the DADA2 (Divisive Amplicon Denoising Algorithm) plugin [43], filtering out any chimeric sequences. Following the removal of resultant singleton amplicon sequence variants (ASVs), taxonomic classification was performed at a confidence threshold of 0.7 using a multinomial naïve Bayes classifier [44] trained on full length 16S rRNA sequences from the SILVA v138 database [45]. All non-bacterial sequences were removed from classified reads, which were then imported into the R environment v 4.3.2 [46].

Using the *decontam* package v 1.22.0 [47], contaminant DNA were identified by performing a prevalence test at a threshold of 0.5, removing any sequences that occur more, by a statistical significance, in extraction controls than in environmental samples. Based on an arbitrary minimum read cut off, samples were filtered by total feature frequency, removing any samples with a read count below 10% of the maximum frequency. Filtered reads were then scaled by ranked subsampling using the *SRS* package v 0.2.3 [48] to achieve a normalised library count across all samples.

### Diversity analysis

Alpha and beta diversity metrics were estimated using the *phyloseq* package v 1.46.0 [49].

Species richness and evenness were first calculated for non-normalised reads based on the indices for Shannon’s Diversity and Pielou’s Evenness, respectively, using the *phyloseq* package. To elucidate whether observed taxonomic diversity differed significantly by substrate type or by site, a Kruskal–Wallis Rank Sum test was performed. This was followed by a pairwise comparison between substrate types per site and within each type across sites using the Wilcoxon Rank Sum Exact test. Both tests were conducted using the *stats* package v 3.6.2 [46].

Using normalised reads, Bray Curtis distances were calculated to assess compositional dissimilarities between the different communities. Variations were then partitioned using a two-way permutational analysis of variance (PERMANOVA) to determine whether substrate type, site, and/or the interaction of the two factors played a significant role in driving the differences observed. An analysis was also conducted to test the homogeneity of multivariate dispersions within groups (PERMDISP). All beta diversity calculations were performed using the *vegan* package v 2.6–4 [50].

### Quantification of PAHs

Only particles sampled after 6 weeks of incubation were screened for a total of 15 PAHs (Additional file 1). PAHs in surrounding waters were not measured as it was not within the scope of this study, the main aim of which was to discern between the sorption patterns of different substrate types and their effects on biofilm communities.

Subsamples of roughly 1 to 3 g were prepared for each sample and PAH extraction performed in several steps. The first extraction was performed using an equal part mixture of methanol and acetonitrile (10 ml each) followed by a second extraction using 10 ml of hexane. Each round of extraction was performed twice. Each time, samples were incubated in their respective solvents within an ultrasonic bath for 30 min. Methanol:acetonitrile and hexane extracts from each sample were then combined and a third extraction performed using liquid–liquid extraction with 10 ml of a water and methanol mixture (2:1). The extract was then evaporated to approximately 1 ml with a gentle nitrogen stream followed by a cleaning step using silica gel. The clean extract was evaporated until completely dry and reconstituted in 1 ml of methanol. Sample extracts were analysed using liquid chromatography coupled to a fluorescence detector and blank measurements performed on pre-incubation, blank particles as negative controls.

### PAH analysis

The distribution of the PAH concentrations measured was assessed using the Shapiro-Wilks test in the *stats* package. After confirming that the dataset did not adhere to a standard Gaussian distribution, values were normalised through log-transformation.

Using the *stats* package, a two-way analysis of variance (ANOVA) was first performed to determine whether substrate type, site, and/or an interaction of the two had a significant effect on the patterns observed of each of the 15 PAHs tested overall. Post hoc pairwise comparisons were then made between substrate types and within each type across the different sites for each of the different PAHs using Tukey’s test.

### Correlation between PAHs and biofilm communities

Potential correlations between PAHs and biofilm communities were explored using the *vegan* package. A Mantel test was first performed on the dissimilarity matrices of communities sampled on week 6 for each of the individual PAHs. Spearman’s correlation coefficient was then calculated to measure the direction and strength of any associations between each PAH and the bacterial orders within week 6 communities. Correlation analyses were further reinforced by a distance-based redundancy analysis (dbRDA) to interpret the relationship between the different PAHs and substrate types across sites based on constrained ordinations. To account for the role of environmental conditions, temperature and salinity measured across sites were also included as variables in the analysis. This was followed by a forward selection step to identify the factors that best explained the variations observed and to improve the accuracy of the model’s prediction.

## Results

### Particles and their distinct PAH profiles along the coast

PE, PS, and stone were incubated at different sites along the Baltic coast to discern between the PAH sorption patterns of different substrates. According to a two-way analysis of variances (ANOVA), PAH sorption patterns were dictated more by substrate type than site or an interaction of the two factors (Additional file 2). All three terms, however, had a significant effect on the sorption of most PAHs tested (*p* < 0.05).

Baseline PAH concentrations were first measured using blank particles of each type. Of the three substrates, total PAH concentrations were the highest on stone blanks, followed by PS, and PE blanks (Additional file 1). Naphthalene and phenanthrene made up the largest proportion of total PAHs detected on the synthetic substrates, whereas stone was dominated by phenanthrene and fluorene.

**Fig. 2:**
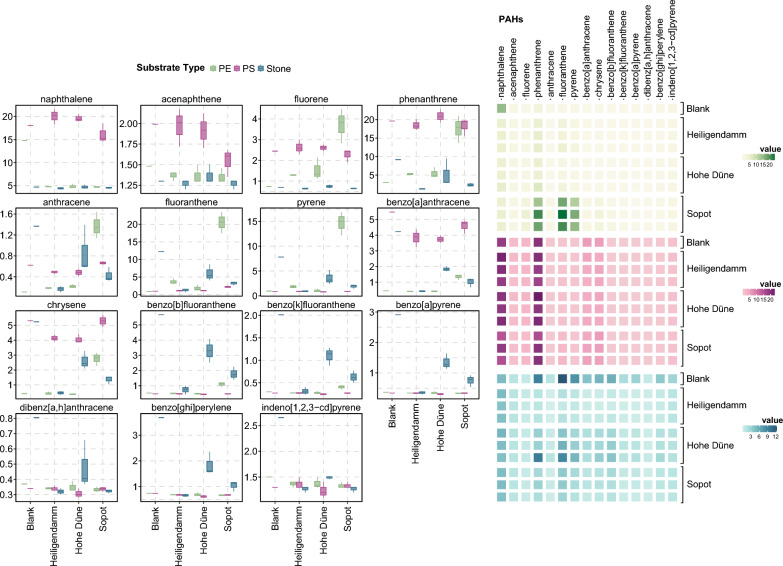
PAH concentrations detected on PE (green), PS (purple), and stone (blue) across the three incubation sites (Heiligendamm, Hohe Düne, Sopot). The distribution of values is illustrated in the form of a box plot for each of the 15 PAHs tested (on the left) and the varying concentrations observed across the different substrate types and sites are contrasted in the form of a heatmap (on the right)

Following incubation in coastal surface waters, each of the substrate types were observed to adhere to sorption patterns distinct from one another. For PS, PAH concentrations remained relatively unchanged after incubation at all three sites (Fig. 2). Similarly, no significant differences were observed in their PAH concentrations between sites (Additional file 3). At the two Mecklenburg Bight sites, total PAH concentrations were the highest on PS. Here, 40% of all tested PAHs were detected at significantly higher concentrations on PS than on the other substrates (*p* < 0.05). Naphthalene and phenanthrene, however, remained the two most dominant fractions on PS.

The profile of PAHs detected on PE also remained relatively unvaried after incubation at the Mecklenburg Bight sites. In Sopot, however, PE sorbed significantly higher concentrations of PAHs (*p* < 0.05), resulting in an almost threefold increase in total PAH concentrations from baseline. This was due in large part to the comparatively greater sorption of more than half of the PAHs tested (namely, fluorene, phenanthrene, anthracene, fluoranthene, pyrene, benzo[a]anthracene, chrysene, and benzo[b]fluoranthene) at this site. Consequently, the highest total PAH concentrations were detected on PE at this site, with significantly higher concentrations of fluorene, fluoranthene, and pyrene found on PE than other substrates.

PAH sorption patterns were most distinct for stone, as the non-plastic counterpart to the two synthetic substrates. Stone was generally marked by higher concentrations of long-chain PAHs and found to leach rather than sorb PAHs in the environment. Across sites, total PAH concentrations on stone fell by 43 – 78% from baseline following incubation.

### Composition of bacterial communities

A total of 116 biofilm and water samples remained following quality control measures, each amounting to a total read count that ranged between 3038 and 33,929 reads. Within biofilm communities, *Alphaproteobacteria*, *Bacteroidia*, and *Gammaproteobacteria* were the most dominant classes across all sites (Additional file 4). *Alphaproteobacteria*, which generally made up the largest fraction across all biofilms, consisted mostly of members belonging to the *Rhodobacterales* order (Additional file 5). *Bacteroidia,* meanwhile, was predominated by the *Flavobacterales* order across all sites.

**Fig. 3:**
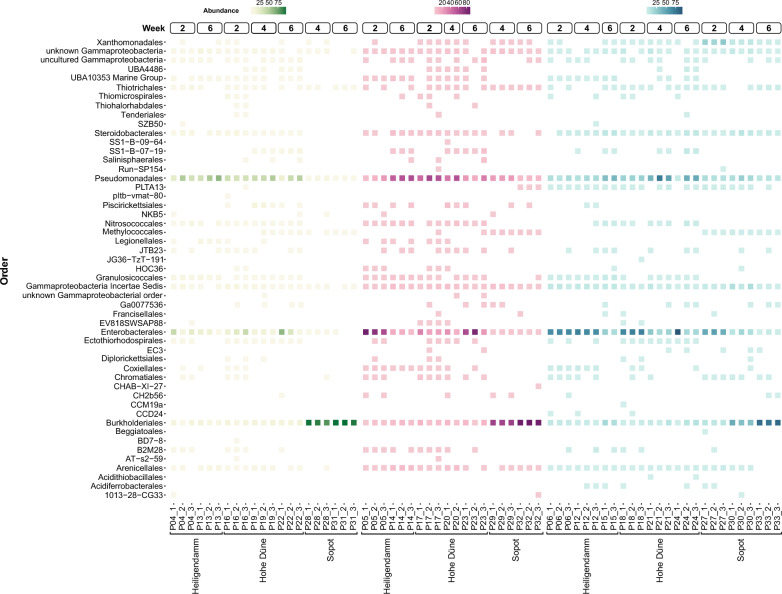
Heatmap showing the relative abundances of Gammaproteobacterial orders detected on PE (green), PS (purple), and stone (blue) on Weeks 2, 4, and 6 across the three incubation sites (Heiligendamm, Hohe Düne, Sopot). The *Gammaproteobacteria* detected across all substrate biofilms were similarly predominated by orders *Pseudomonadales* and *Enterobacterales* in Heiligendamm and Hohe Düne. In Sopot, conversely, *Burkholderiales* made up the most dominant proportion of the *Gammaproteobacteria* class across all substrate types

Variations in biofilm communities by site were most pronounced within the *Gammaproteobacteria* class (Fig. 3). In the Mecklenburg Bight, *Enterobacterales* and *Pseudomonadales* were the two most dominant orders of *Gammaproteobacteria* detected across biofilms. In Sopot, however, *Burkholderiales* made up the largest proportion. The latter observation was particularly evident among PE biofilms, where *Burkholderiales* made up more than 90% of *Gammaproteobacteria* detected at this site.

While *Alphaproteobacteria* made up the largest proportion of free-living (0.22 µm) waterborne communities, more site-specific patterns were observed among particle-associated (3.0 µm) waterborne communities. In Hohe Düne, the latter was predominated by the *Cyanobacteriia* class, while *Bacteroidia* was most dominant at the other two sites.

### Distinctions in the diversity and composition of biofilm communities

As was the case with PAHs, community composition was found to be most strongly shaped by substrate type, followed by incubation site, and the interaction of the two (Additional file 6). In ordination space, the most distinct compositional differences were observed between biofilm and waterborne communities (Axis 1, 28.2%), followed by site differences (Axis 2, 12.7%) (Additional file 7). All communities (biofilm and waterborne) differed significantly from each other in their composition at all sites, except for those on PE and PS, between which no significant differences were observed (*p* < 0.05, Additional file 8). Significant compositional variation between sites was also observed within communities associated to each sample type. It is important to note, however, that the significance of observed compositional differences should be interpreted conservatively as significant patterns of heterogeneity were also detected within all groups (PERMDISP *p* < 0.05). To visually assess within-group dispersions, a PCoA plot of only the particle samples was drawn, confirming that these variations did not mask those observed between groups (Additional File 9).

Species richness and evenness were greater among biofilm communities than surrounding waterborne communities (*p* < 0.05, Additional files 10 and 11). Biofilm communities sampled from synthetic and stone substrates also differed significantly from each other at two of the three sites (Heiligendamm and Sopot), with stone communities observed to be richer and more even. No significant differences were observed, however, between the overall diversity of PE and PS communities except at the Sopot site. Here, the richness and evenness of all biofilm communities were the lowest and differed significantly from each other. PE biofilms, however, were the least diverse of all sample types.

### Correlation between PAH sorption patterns and biofilm communities

In assessing the role that PAHs potentially play in shaping MP biofilms, correlation and redundancy analyses were conducted on all week 6 biofilm communities across all sites.

Detected PAH concentrations were found to correlate positively to the observed community composition overall (R = 0.289; *p* = 0.001). Mantel tests conducted on individual PAHs and community dissimilarity matrices revealed that of the 15 PAHs tested, 7 shared significant correlations with observed community composition (*p* < 0.05, Additional file 12). These comprised both short-chain PAHs (anthracene, fluorene, phenanthrene, fluoranthene, pyrene) and long-chain PAHs (benzo[b]fluoranthene, benzo[k]fluoranthene).

Constrained ordinations revealed that overall PAH concentrations and environmental conditions (temperature and salinity) explained 77.7% of the variation observed among bacterial communities (Additional file 13**)**. Of the 17 variables tested (temperature, salinity, PAHs), 6 were identified through forward selection to be more ‘statistically important’, explaining 69.9% of the total variation observed. In descending order of effect size (F-value), these consisted of temperature, salinity, fluorene, fluoranthene, anthracene, and phenanthrene (Additional file 14).

**Fig. 4:**
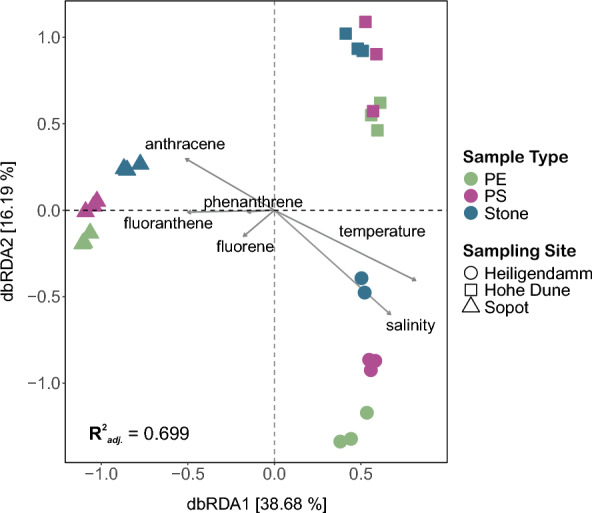
Distance-based Redundancy Analysis (dbRDA) elucidating the relationship between forward selected explanatory variables (temperature, salinity, PAHs) and community dissimilarities observed among the different particle substrates (PE, PS, stone) in Week 6 across the different sites based on the Bray–Curtis index. Proportion of explained variation are presented along the axes while relationships and their strength are illustrated by the superimposed arrows and their length, respectively. The greatest variance among substrate biofilms is captured along the first axis (dbRDA1, 38.68%) between communities from Sopot and the other two sites (Heiligendamm and Hohe Düne), with selected variables explaining 69.9% of all differences observed (R^2^_*adj.*_,)

The greatest proportion of variation (38.68%) was observed along the first axis, between communities from Sopot and the two Mecklenburg Bight sites (dbRDA1, F = 32.17, *p* = 0.001) (Fig. 4). The distinction was most strongly explained by temperature and salinity, which appeared to correlate negatively to the similarly significant effects of fluorene, fluoranthene, anthracene, and phenanthrene detected on the different substrates across sites. Along the second axis, a comparatively lesser separation was also observed between the different substrate biofilms and between communities from the Mecklenburg Bight sites (dbRDA2; F = 13.5, *p* = 0.001).

The relationships between specific PAHs and members of the biofilm communities were further explored through measures of Spearman’s rank correlation. The distinct profiles of PAHs detected on the different substrate types were found to share strong positive relationships with specific bacterial taxa.

On PE, fluorene, phenanthrene, and anthracene (3-ring PAHs) correlated strongly with Alpha- and Gammaproteobacterial orders *Rhodobacterales*, *Holosporales*, *Burkholderiales*, and *Micavibrionales* (Additional file 15). These orders were detected in higher relative proportions in Sopot, coinciding with elevated concentrations of fluorene, phenanthrene, and anthracene observed on PE here. *Burkholderiales*, in particular, constituted a comparatively higher proportion of PE communities at this site than in the Mecklenburg Bight (Fig. 3; Additional file 5). Positive correlations were observed between *Burkholderiales* and many of the PAHs detected on PE (fluorene, phenanthrene, anthracene, fluoranthene, pyrene, benzo[b]fluoranthene, benzo[k]fluoranthene). Conversely, 4-ring PAH, fluoranthene shared the strongest correlations to bacterial orders *Holosporales*, SAR11 clade, *Flavobacteriales*, and *Thiotrichales*.

On PS, these PAHs correlated to slightly different taxa than on PE. Fluoranthene and anthracene shared strong positive relationships with similar bacterial orders belonging to *Gammaproteobacteria* (*Methylococcales, Burkholderiales, Xanthomonadales, Thiotrichales, PLTA13*), *Bacteroidia* (*Cytophagales*), and *Parcubacteria* (*Candidatus Kaiserbacteria*). Fluorene and phenanthrene, conversely, shared strong correlations to orders of *Planctomycetes* (Planctomycetales) and *Gammaproteobacteria* (*Salinisphaerales*)*.*

On stone, long-chain PAHs, benzo[b]fluoranthene, benzo[k]fluoranthene, benzo[ghi]perylene, and benzo[a]pyrene, were detected in highest concentrations of all substrates. Detected in highest concentrations in Hohe Düne, these PAHs shared a strong positive correlation to the same bacterial orders *Ectothiorhodospirales* (*Gammaproteobacteria*), *Planctomycetales* (*Planctomycetes)*, and uncultured *Desulfobacterota,* all of which were similarly detected in greatest proportion at this site.

## Discussion

### Substrate type influences PAH sorption behaviour of MP

Concerns over the widespread distribution and impact of emerging pollutants on surrounding ecological systems and human health are long-established. The risks that MP pose, as one such pollutant, stem not only from their durability and dispersibility but also from their capacity to sorb persistent pollutants in the environment. Numerous studies have been conducted on MP sorption of PAHs due to the well-established ubiquity of the latter throughout aquatic ecosystems [8, 9, 12, 13, 51–53]. For instance, in a monitoring effort by the International Pellet Watch (IPW) in 2005, PE pellets were sampled from 193 locations worldwide and analysed for POPs. While PE pellets sampled from remote locations contained, on average, levels of POPs several orders of magnitude lower than those from industrial regions, in some cases POP concentrations on PE from both environments, pristine and polluted, were comparable [54]. The combined impact of these toxic compounds, however, remains comparatively underexplored. Investigations on the mechanics and driving factors behind the sorption process [8, 9, 13, 53] determined the sorption behaviour of plastic polymers to be dependent on a combination of factors. While also conditional upon environmental parameters, such as temperature, salinity, or trophic state, sorption is largely dictated by the attributes of the substrate [52, 55, 56]. This was concurrent with the findings of our study, where PAH sorption patterns were shaped more by substrate type (PE, PS, stone) than incubation site. This distinction between substrate types was most profound when comparing between stone and synthetic substrates.

### PAH sorption patterns of PE and PS

PE and PS, in comparison to stone, were characterised more by the sorption of short-chain PAHs (≤ 4 rings). As short-chain, low molecular weight PAHs are less hydrophobic, they generally achieve sorption equilibrium on plastic polymers more quickly than their larger, heavier analogues [6, 57, 58]. It is important to note, however, that this is also contingent on factors such as polymer type, polarity, or surface chemistry. Upon exposure to aquatic environments, plastic surfaces become weathered by UV irradiation and the mechanical forces of nature. In addition to an increased surface area for sorption, weathering results in the formation of a greater number of oxygen-containing functional groups that increase the hydrophilicity of the plastic surface [58, 59]. Weathered plastics, in turn, possess a greater sorption capacity for more hydrophilic molecules [59, 60] such as short-chain PAHs. Long-chain, high molecular weight PAHs, due to their highly hydrophobic nature and consequent lack of solubility, are thus more often observed to be enriched on naturally occurring particulate matter [14, 61]. This was observed in our study with stone, which was marked by PAHs with 5 or more rings.

In our study, stone was found mostly to leach rather than sorb PAHs in the environment. Stone pellets were used in this study to serve as a non-plastic control to distinguish any unspecific particle effects from plastic-specific effects. As aquaria stones were used, however, we acknowledge that the baseline PAH concentrations and leaching behaviour observed of stone in our study do not necessarily reflect the behaviour of stones found in nature. Any patterns observed of the stones used in this study, therefore, should not be taken to represent the behaviour of natural stone, as the pellets used were pre-processed. We speculate further, based on the detection of PAHs on blank stone pellets and the general leaching behaviour observed during incubation, that the majority of PAHs detected on stone were the industrial residues of the manufacturing process.

At two of the three sites (Heiligendamm and Hohe Düne), total PAH concentrations were the highest on PS, with no significant variation observed in their PAH profiles before or after incubation. This leads us to posit that detected PAHs likely stemmed from production residues or the polymer itself. In fact, PS has been shown to produce the greatest PAH emissions of a range of different plastic polymers [62]. Upon the depolymerisation of PS, Wheatley et al. found naphthalene and phenanthrene to be among the most prominent PAHs detected, along with methylnaphthalene, methylfluorene, and biphenyl. Similarly, in our study, total PAH concentrations detected on blank PS particles were twice that of blank PE particles, with naphthalene and phenanthrene making up the two largest proportions of all the PAHs tested. These observations thus underpin our thesis that the PAH profile detected on PS in our study likely stemmed from manufacturing residues or the polymer itself.

In Sopot, the highest total PAH concentrations were observed on PE. Situated within the Gulf of Gdańsk, the Sopot site is characterised by strong riverine inputs of freshwater on the surface [37] and as such, waters here were the least saline of the three incubation sites. Receiving influences from nine major rivers, this catchment is characterised by heavy pollution loads [37, 63]. Overall PAH levels were reported by Naumann and colleagues to be higher in this area than in the Mecklenburg Bight [64]. In the winter of 2019, fluorene, fluoranthene, and phenanthrene were found to make up the largest proportion of the 15 PAHs tested within surface waters across both regions. These PAHs amounted to a total of 4436 ng/m^3^ in the Mecklenburg Bight (∑PAH = 6022 ng/m3) and 6389 ng/mg^3^ in the Central Baltic Sea (∑PAH = 9486 ng/m^3^). In their study, the three short-chain PAHs were found to predominate the dissolved fraction of surface waters while long-chain PAHs were more prevalent in particle-associated fractions (namely indeno[1,2,3-cd]pyrene, benzo[b]fluoranthene, and benzo[ghi]perylene). In our study, significantly higher concentrations of fluorene, fluoranthene, and phenanthrene were also detected on PE compared to PS or stone at the Sopot site.

Differences between the sorption behaviour observed of PE and PS are likely attributed to their contrasting physical properties. PE, considered a rubbery polymer, has a higher diffusivity and thus greater sorption rate than PS [8, 9]. The latter, categorised as a glassy polymer, sorbs in a non-linear fashion, with chemicals more likely to partition into nano-sized pores [65–67]. PS, although reported to have a sorption capacity comparable to that of PE, can thus take up to a year to reach sorption equilibrium [8, 9]. It is possible that concentrations of PAHs sorbed by the different polymer types might have been comparable had our incubation time been extended beyond 6 weeks. Considering the specific sorption characteristics of PE, however, these polymers might serve as a better proxy for surrounding environmental conditions.

### PAH sorption patterns potentially play a role in shaping MP biofilms

All substrate biofilms were similarly dominated by *Alphaproteobacteria*, *Bacteroidia*, and *Gammaproteobacteria*. These bacterial classes are described as typical members of aquatic biofilm communities and likewise are commonly reported to dominate microplastic surfaces in these environments [68–71]. The composition and diversity of biofilm communities were found to correlate significantly with the respective PAH profiles observed of the different substrate types. The strong correlation patterns detected among specific taxa and individual PAHs across sites strongly indicate a response among biofilm communities to the PAH sorption patterns observed. These correlations, however, are likely conditional upon a wide range of factors, and further investigations are required to confirm the suggested relationship between PAHs and biofilm members. Among these explanatory factors, we posit environmental conditions to play an important role. Through a redundancy analysis, temperature and salinity were found to be significant drivers of variations observed among biofilm communities across sites, alongside 4 short-chain PAHs. As these environmental parameters shared inverse correlations to the 4 PAHs, we hypothesise the observed variations among biofilm communities to potentially be the product of environmentally driven sorption patterns, resulting in the prominence of select PAHs and therewith specific taxa. In previous studies, temperature and salinity have been demonstrated to have a significant effect on particle sorption of PAHs [72, 73]. Low temperatures and/or high salinities were found to reduce the solubility of PAHs, resulting in their greater sorption on particle surfaces [73]. While waters in Sopot were the least saline of the three sites, they were also the coldest. Additionally, this site being partially sheltered, and thus stagnant in nature, retains greater anthropogenic effluents than the Mecklenburg Bight [37, 64]. It is therefore possible that a combination of low temperatures and higher PAH exposure at this site distinguished biofilm communities in Sopot from those in the other two sites.

### PE-associated correlations

Of the 15 PAHs tested, fluorene, anthracene, fluoranthene, and phenanthrene were identified through a redundancy analysis as the strongest drivers of compositional differences observed between PE biofilms across sites. These PAHs corresponded positively to common orders *Micavibrionales*, *Thiotrichales*, *Holosporales,* and *Burkholderiales.* Higher proportions of these bacterial orders were detected on PE at Sopot compared to other sites, coinciding with elevated levels of fluorene, fluoranthene, anthracene, and phenanthrene observed on this substrate. Apart from *Holosporales*, these Alpha- and Gammaprotebacterial orders are known to possess the metabolic capabilities for PAH utilization [14, 17, 74, 75]. *Burkholderiales*, which was predominated by the *Comamonadaceae* family, particularly in Sopot, are known hydrocarbon utilisers reportedly enriched upon exposure to PAHs [14, 76]. While similar reports have been made of *Thiotrichales* in PAH-contaminated seawaters, there is currently no evidence that members directly utilize hydrocarbons as a substrate [77, 78]. Rather, research suggests that these bacteria utilize the carbonic by-products of PAHs [77]. In our study, the order comprised the *Thiotrichaceae* family, members of which have been reported to encode for the benzoyl-CoA pathway involved in the catabolism of a wide range of monocyclic aromatic compounds [79]. As obligate predatory bacteria, *Micavibrionales* were posited by Nikolova and colleagues to be grazing on oil-degrading bacteria in bloom, resulting in their reported enrichment in certain hydrocarbon-polluted environments [74]. Little is known about *Holosporales*, which in our study consisted of the *Holosporaceae* family, beyond the fact that they primarily form obligate, endosymbiotic relationships with eukaryotes [80]. Without deeper investigation, we can therefore only speculate on a potential syntrophy between *Holosporaceae* and other members of the community in relation to PAHs.

### PS-associated correlations

Fluorene, fluoranthene, anthracene, and phenanthrene also played a potential role in shaping PS biofilms across sites. As was the case with PE, anthracene and fluoranthene corresponded strongly to the four aforementioned orders (*Micavibrionales*, *Thiotrichales*, *Holosporales,* and *Burkholderiales*) in addition to the methanotrophic order, *Methylococcales* (predominated by *Methylomonadaceae* family), and the hydrocarbonoclastic order, *Xanthomonadales* (predominated by *Xanthomonadaceae* and *Rhodanobacteraceae* families) [81, 82]. Fluorene and phenanthrene, on the other hand, correlated strongly to *Salinisphaerales*, which in our study comprised the *Solimonadaceae* family, whose members have a reported capacity for hydrocarbon degradation [83]. These predominant families, observed to putatively correlate to the profile of PAHs sorbed, were generally found either exclusively or in greater proportions within biofilm communities than surrounding waters.

### Potential effects of PAH sorption patterns on biofilm diversity

We posit this allegedly selective effect of PAHs on substrate biofilms to also play a role in the species diversity patterns observed. Communities sampled from Sopot (biofilm and waterborne) were less diverse than those in other sites, with PE communities being the least diverse of all the sample types here. While we acknowledge the potential impact of surrounding environmental conditions, the lower diversity observed of PE communities might additionally be linked with the elevated levels of PAHs sorbed by PE at this site. It has in fact been suggested that elevated levels of different PAHs in combination can decrease the overall diversity of impacted bacterial communities [19, 84]. This was also demonstrated in a lab-based experiment by Fernández-Juárez and colleagues, where plastic biofilms were inhibited in their growth when exposed to fluoranthene [33]. Further studies are required, however, to confirm any causal relationship between PAHs and the diversity of MP communities.

The potential associations observed in our study between PAH sorption patterns and the dynamics of biofilm communities do, however, illustrate the importance of exercising caution when interpreting the membership of MP biofilms. The presence of hydrocarbon utilisers within MP biofilms should not be mistaken as an indicator for polymer specificity or plastic degradation without additionally considering the role of compounds sorbed by the substrate. Our findings serve as evidence for the necessity to consider not only the substrate in itself but all compounds accessory to it, sorbed or leached, when attempting to understand their interactions and effects on surrounding microbial communities. Beyond that, we offer early insights into the potential role PAHs play in shaping the membership, structure, and diversity of MP biofilms, as evidenced by the strong correlations found between specific PAHs and reportedly hydrocarbonoclastic taxa.

## Conclusions

While the impact of environmental pollutants has always been the subject of extensive research, their intersection and combined effects on surrounding biomes are arguably less explored. With the addition of MPs to a growing list of environmental contaminants, further complexities are introduced to an already convoluted network of pollution. We highlight with our study the necessity to rethink this network of pollutants throughout aquatic ecosystems due to the highly interactive nature of these synthetic polymers in the environment. The detection of MPs laden with PAHs across different environments worldwide, pristine or industrial, clearly demonstrates the capacity of these synthetic polymers to behave as long-range transport vectors.

The interactions between PAHs and MPs are further convoluted by the interactions of the latter with surrounding communities of heterogenous bacteria. As microbial communities play critical roles in the regulation of many fundamental ecological processes, it is important to investigate how these communities might be altered or impacted by this interplay of pollutants. We show with our study that within pelagic environments, biofilms can potentially be shaped by the specific profile of PAHs that are sorbed and/or leached by these synthetic polymers. The findings highlight the importance of further investigations into the extent and impacts of these complex interactions on fragile ecological systems. The limitations of this study should also be addressed in future work. In order to confirm any causal relationship between contaminant interactions and associated microbial communities, assessments of the contaminant levels in surrounding waters and lab-based experiments on the metabolic responses of biofilm communities to PAH exposure are necessary. To fully comprehend how MPs interact and alter ecosystems, microbial or otherwise, the entire substrate must be considered, which includes all chemicals integrated into the polymeric matrix, albeit elemental or sorbed, as it travels through various environmental compartments.

## Supplementary Information

Additional file 1.

Additional file 2.

Additional file 3.

Additional file 4.

Additional file 5.

Additional file 6.

Additional file 7.

Additional file 8.

Additional file 9.

Additional file 10.

Additional file 11.

Additional file 12.

Additional file 13.

Additional file 14.

Additional file 15.

## Data Availability

The datasets generated and/or analysed during the current study are available in the GitHub repository here: https://github.com/jessicaxsong/CloseEncounters. 16S rRNA sequences generated are deposited in the European Nucleotide Archive at EMBL-EBI under accession number PRJEB85453.
