# Supplementary material for: Close encounters on a micro scale: microplastic sorption of polycyclic aromatic hydrocarbons and their potential effects on associated biofilm communities
Source: Environ Microbiome. 2025 Jul 8;20:84. doi: 10.1186/s40793-025-00747-w (PMC12239331; doi:10.1186/s40793-025-00747-w)
Supplement: Supplementary file 4 — Additional file 4. [file 40793_2025_747_MOESM4_ESM.pdf]

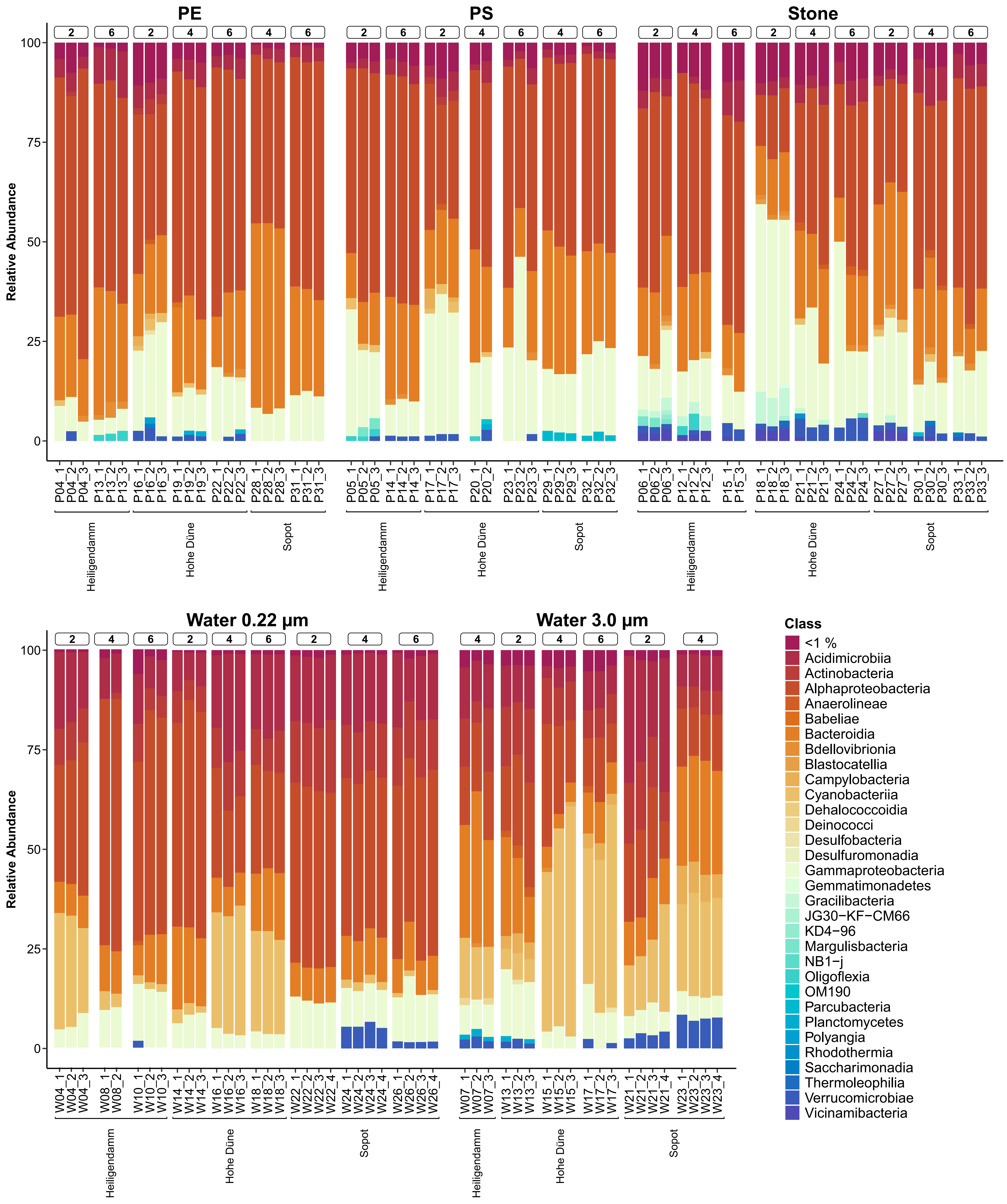

Additional file 4 Stacked barplot showing the relative abundances of bacterial classes detected within the different communities (biofilm and waterborne) across the different sites.
