# Supplementary material for: Close encounters on a micro scale: microplastic sorption of polycyclic aromatic hydrocarbons and their potential effects on associated biofilm communities
Source: Environ Microbiome. 2025 Jul 8;20:84. doi: 10.1186/s40793-025-00747-w (PMC12239331; doi:10.1186/s40793-025-00747-w)
Supplement: Supplementary file 7 — Additional file 7. [file 40793_2025_747_MOESM7_ESM.pdf]

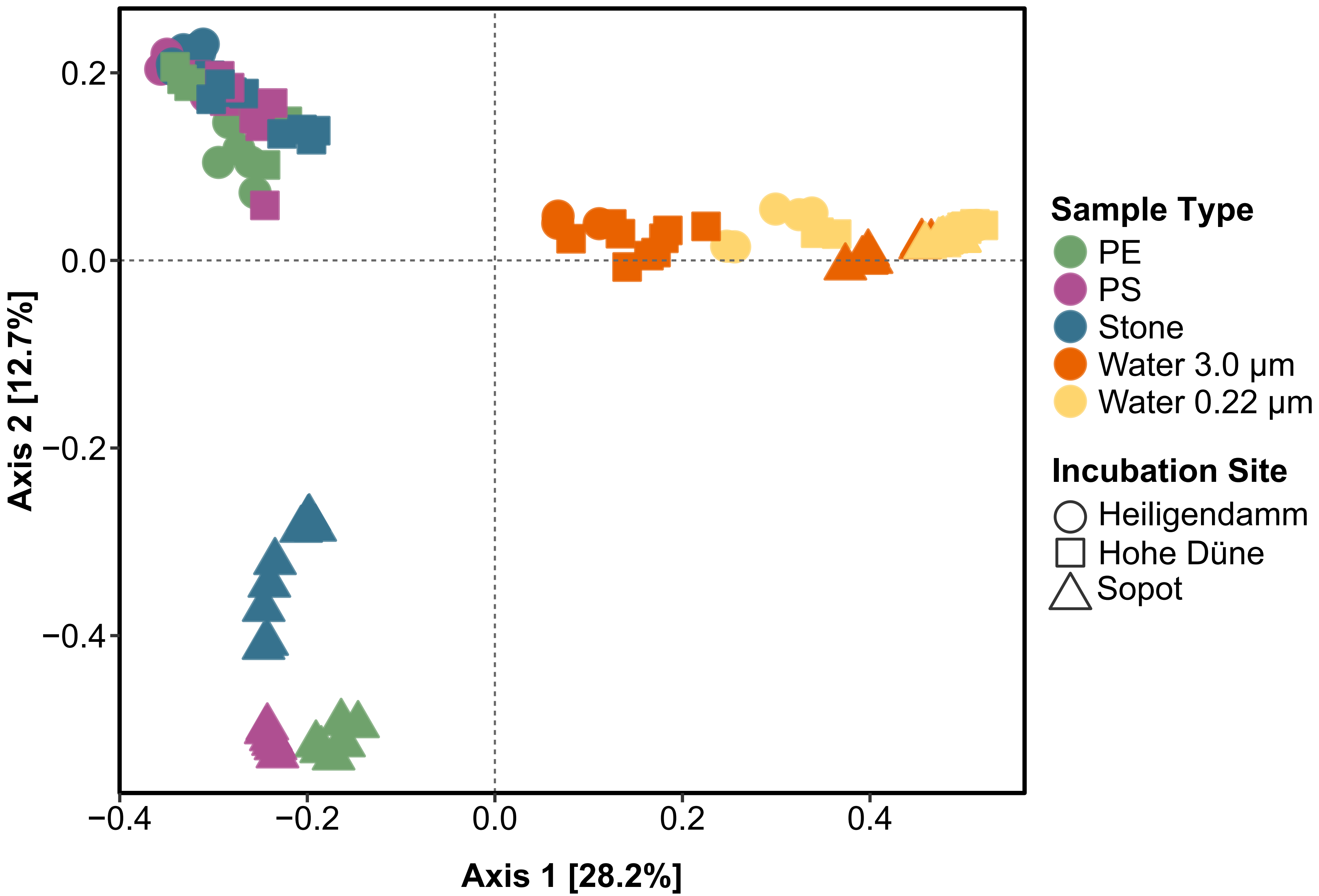

Additional file 7 Principal Coordinate Analysis (PCoA) displaying the structural dissimilarities observed between the different sample types (PE, PS, stone, Water 3.0 µm (particle-associated waterborne communities), Water 0.2 µm (free-living waterborne communities)) across the different sites based on the Bray-Curtis index.
