# Supplementary material for: Close encounters on a micro scale: microplastic sorption of polycyclic aromatic hydrocarbons and their potential effects on associated biofilm communities
Source: Environ Microbiome. 2025 Jul 8;20:84. doi: 10.1186/s40793-025-00747-w (PMC12239331; doi:10.1186/s40793-025-00747-w)
Supplement: Supplementary file 9 — Additional file 9. [file 40793_2025_747_MOESM9_ESM.pdf]

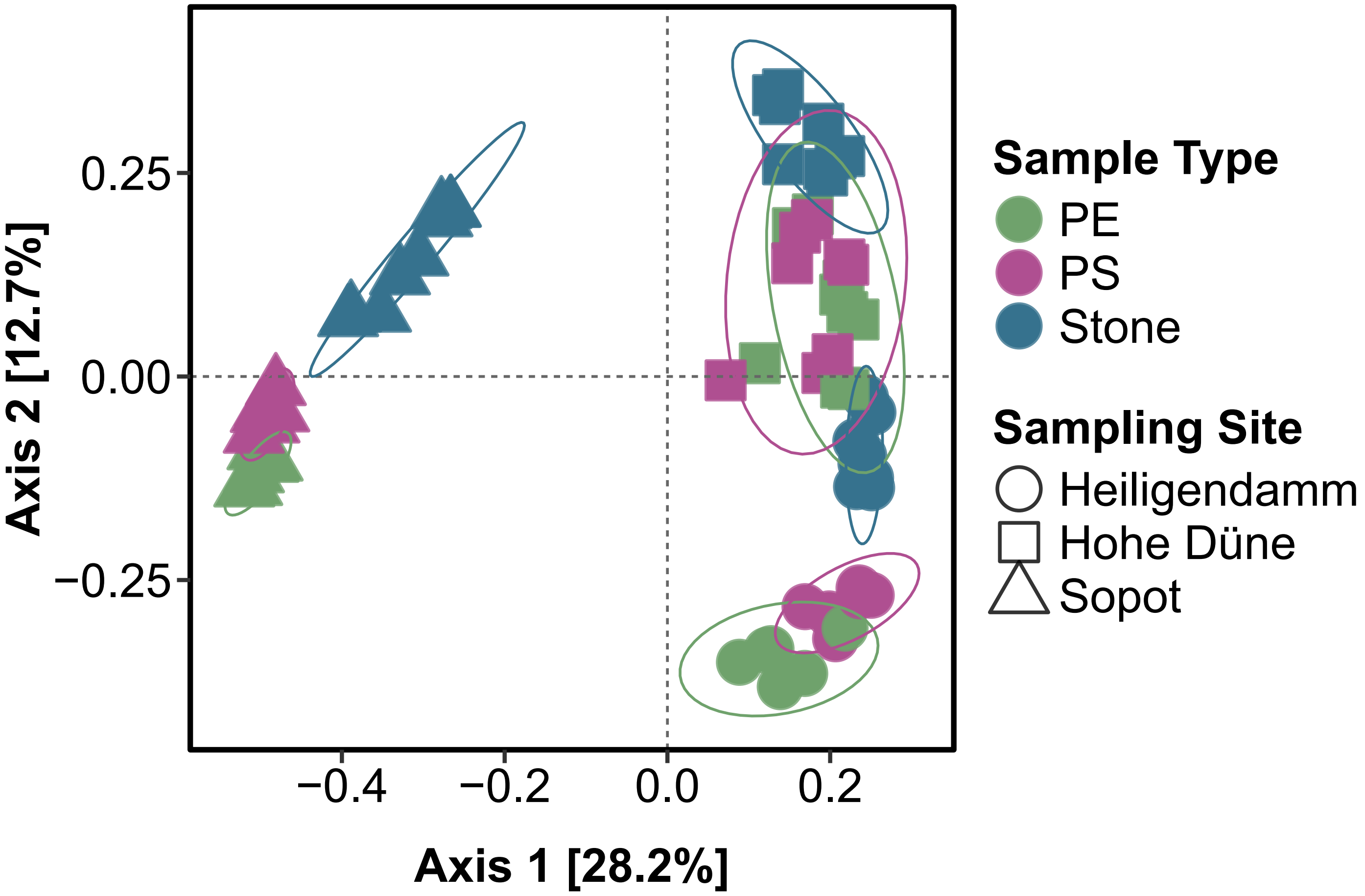

Additional file 9 Principal Coordinate Analysis (PCoA) displaying the structural dissimilarities observed between the different particle types (PE, PS, stone) across the different sites based on the Bray-Curtis index. Ordination of particle biofilms serve to visualise dispersions within the different groups, demonstrating that within-group variationsdid not mask any pattern of variation observed between groups.
