# Supplementary material for: Close encounters on a micro scale: microplastic sorption of polycyclic aromatic hydrocarbons and their potential effects on associated biofilm communities
Source: Environ Microbiome. 2025 Jul 8;20:84. doi: 10.1186/s40793-025-00747-w (PMC12239331; doi:10.1186/s40793-025-00747-w)
Supplement: Supplementary file 10 — Additional file 10. [file 40793_2025_747_MOESM10_ESM.pdf]

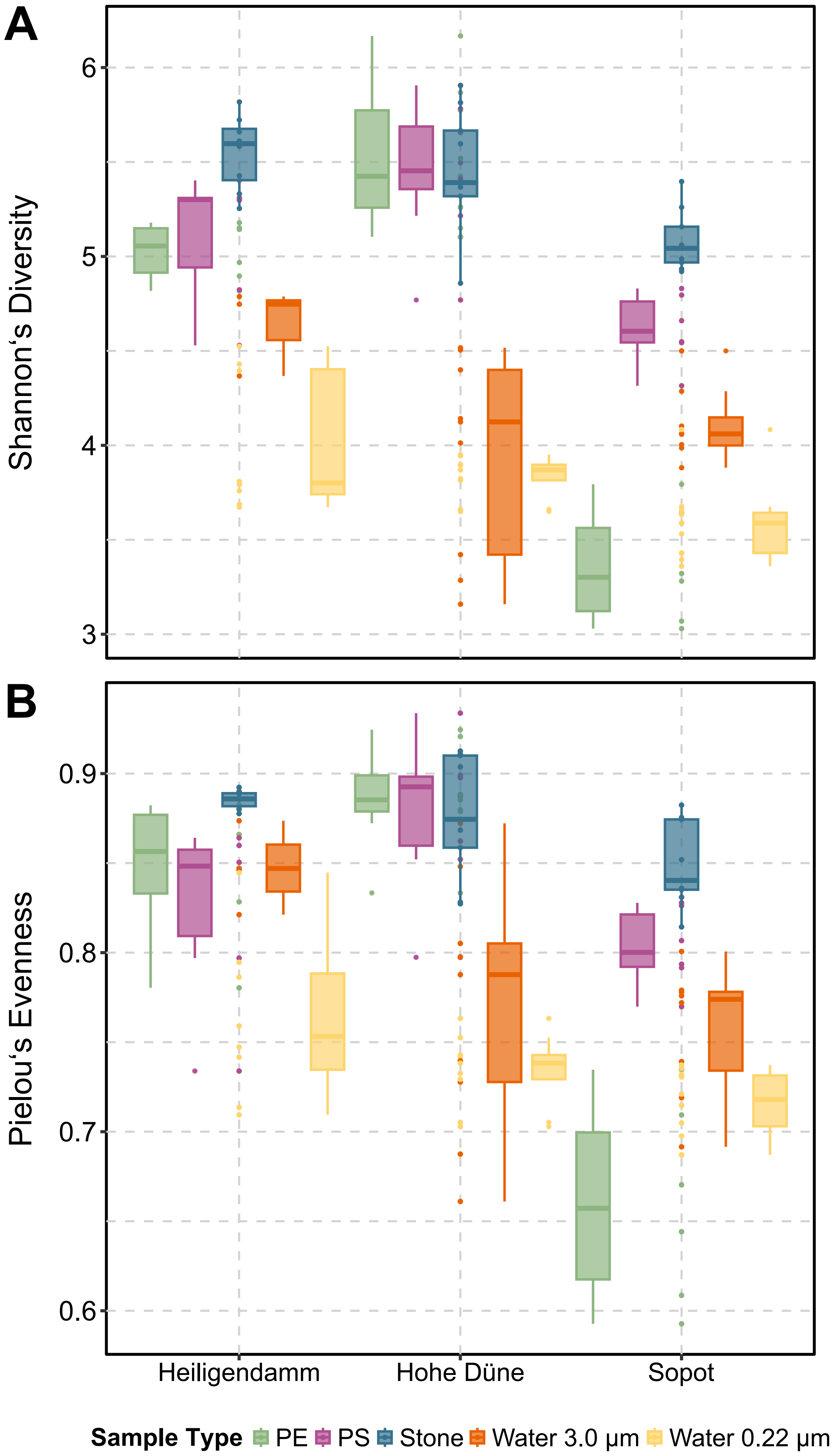

Additional file 10 Boxplots displaying the species diversity (**A**) and evenness (**B**) of communities observed across the different sample types and sites based on Shannon‘s Diversity and Pielou‘s Evenness indices, respectively.
